# Stable Consciousness? The “Hard Problem” Historically Reconstructed and in Perspective of Neurophenomenological Research on Meditation

**DOI:** 10.3389/fpsyg.2022.914322

**Published:** 2022-05-26

**Authors:** Stephan Schleim

**Affiliations:** Theory and History of Psychology, Heymans Institute for Psychological Research, Faculty of Behavioural and Social Sciences, University of Groningen, Groningen, Netherlands

**Keywords:** consciousness, hard problem, neurophenomenology, meditation, introspection, Wilhelm Wundt, phenomenological psychology

## Abstract

Finding a scientific, third-person explanation of subjective experience or phenomenal content is commonly called the “hard problem” of consciousness. There has recently been a surge in neuropsychological research on meditation in general and long-term meditators in particular. These experimental subjects are allegedly capable of generating a stable state of consciousness over a prolonged period of time, which makes experimentation with them an interesting paradigm for consciousness research. This perspective article starts out with a historical reconstruction of the “hard problem,” tracing it back to Gottfried Wilhelm Leibniz and Emil du Bois-Reymond in the 18th and 19th century, respectively, and the problem of introspection as already acknowledged by Wilhelm Wundt in the 19th century. It then discusses the prospects of research on long-term meditators from a contemporary perspective and with respect to the neurophenomenological research program already advocated by Francisco J. Varela.

## Introduction

Finding the biological basis of consciousness is sometimes considered as one of the major unsolved puzzles of contemporary science (Miller, 2005). However, philosophical arguments commonly subsumed as the “hard problem” of consciousness question the possibility of this endeavor, at least with respect to *subjective experience* (Chalmers, 1995). The issue is even more complicated by the lacking consensus in both psychology and neuroscience on what precisely is to be explained (the so-called *explanandum*) and what an explanation would look like (the *explanans*). For example, Northoff and Lamme (2020) distinguish eight different explanatory frameworks with different views on the *explanandum*, different experimental approaches, and different findings. Another recent review similarly distinguishes even nine modern models for explaining consciousness (Signorelli et al., 2021). That such distinctions matter empirically is illustrated by the example that researchers pursuing the Global Neural Workspace Theory commonly identify regions in the *prefrontal* cortex as candidates for the minimally sufficient neural correlate of consciousness, while scientists following the Integrated Information Theory commonly find *posterior* brain areas to be more active in their experiments (Koch et al., 2016; Northoff and Lamme, 2020).

The aim of this perspective is not to propose yet another framework or to unify the already existing accounts (but see Wiese, 2020). Instead, I first summarize the historical precursors of the presently known “hard problem.” It turns out that the core of the argument has already been formulated by Gottfried Wilhelm Leibniz (1646–1716) in the 18th and Emil du Bois-Reymond (1818–1896) in the 19th century (Leibniz, 1714/2014; Du Bois-Reymond, 1872).^1^ This is then related to Wilhelm Wundt’s (1832–1920) view of experimental psychology and the problem of introspection, particularly the lacking stability of consciousness and the impossibility to observe it without changing it (Wundt, 1888). Decades later, John B. Watson (1878–1958) and other behaviorists banned consciousness from scientific investigation because of its (alleged) vagueness and the unavailability of reliable instruments (Watson, 1913). The arguments gathered thus far will, secondly, be discussed with respect to Francisco J. Varela’s (1946–2001) neurophenomenological research program (Varela, 1996). In particular, recent reviews of meditation research and one exemplary study will be discussed with respect to the possibility of overcoming the “hard problem” by stabilizing consciousness in deep meditation (Winter et al., 2020).

## The “Hard Problem” Historically

The common reference for the “hard problem” of consciousness has become David Chalmers’s article “Facing Up to the Problem of Consciousness” (Chalmers, 1995).^2^ There he distinguished rather “easy” problems to scientifically explain *cognitive functions* (like the ability to discriminate, categorize, and react to environmental stimuli or the integration of information) from explaining *subjective experience*, the “something it is like” to be a conscious organism (see also Nagel, 1974). As described in the introduction, recent reviews of psychological and neuroscientific accounts of consciousness neither agree on the *explanandum* nor the *explanans* of consciousness. Signorelli et al. (2021) conclude in particular that before explaining consciousness, “one needs first to be precise about what it would mean to ‘explain’ something.” Arguments of this kind are *negative*: We don’t know precisely what has to be explained or what an explanation should look like. Can we also find a *positive* argument for why consciousness might be scientifically inexplicable–or at least so hard to explain?

A classic source to look for is Leibniz’s *Monadology* (1714/2014). In § 17 of his major philosophical work, he proposed a thought experiment: Imagine that there were a machine that could think, feel, and perceive just like yourself. Now also imagine to increase it in size such that you could walk around in it like in a mill. If you did, you would see mechanical parts working on each other–like cogwheels and a millstone. But nothing of that mechanism, none of these activities and motions, would explain a perception. For Leibniz, perceptions and the like were properties of *the whole* which cannot be explained by properties of its parts; in modern terms we might say that he denied the possibility of a *reductive* explanation and considered consciousness an emergent phenomenon (Stephan, 2006). Transferred into our time, we might imagine a living human brain increased in size such that we could walk around in it like in a factory (Bieri, 1995). By looking at the neurons and other cells–their synapses, the molecules, and the like–we would see, in analogy to Leibniz’s thought experiment, nothing to explain subjective experience. In a way, brain scanning and other techniques were developed to allow precisely this, to investigate activities of cells and neural networks in the microscopic world of the brain (Schleim and Roiser, 2009). But all we see are accompanying neurophysiological processes, not consciousness itself. Here we must be careful, though, to not beg the question: The argument is supposed to show that there is no reductive explanation for consciousness. One might say, though, that Leibniz’s thought experiment is not a real argument, but an appeal to our imagination or intuition; Dennett might call it an “intuition pump” (Dennett, 1993; Brendel, 2004).

In the 19th century, some 160 years after Leibniz, physiologist du Bois-Reymond gave a couple of influential lectures on the limits of scientific knowledge. In one of them, he picked up Leibniz’s thought experiment and developed it further: Imagine Laplace’s Demon, an intelligence knowing all scientific facts of the world. That super-intelligence would know everything of the atoms moving while you are feeling pain, lust, taste something sweet, smell a rose, hear a tone, see a color, and the like. Du Bois-Reymond (1872) only saw two possibilities: Either the atoms themselves were already conscious, which would not provide an explanation; or their motions and activities wouldn’t explain consciousness. The brain of a (dreamlessly) sleeping person wouldn’t pose a riddle to the Demon, but as soon as the person woke up and became conscious that would change. Again, as with Leibniz, the thought experiment is intended to support the intuition that a full mechanistic explanation of the nervous system cannot explain consciousness. We thus must again be careful not to beg the question.

This situation is different on Wilhelm Wundt’s account, the founder of the first laboratory for psychological experimentation. Wundt sharply distinguished psychology as an experimental science from a broader perceived cultural psychology (Wundt, 1888; de Freitas Araujo, 2016). The former would require *observation* and not just *perception*. To illustrate this difference, Wundt compared somebody’s perception of a lightning with the case of a botanist accidentally discovering an interesting plant on a hike. Wundt was aware of what is commonly known as the problem of introspection, although he himself didn’t use that term (de Freitas Araujo, 2016). That is, consciousness is changing all the time and paying attention to itself also changes it; furthermore, recalling a conscious experience from memory carries the risk of missing important features of the original process or of inventing some that weren’t originally there. By contrast, the botanist’s plant remains stable and can be observed in many different ways. This explains why Wundt preferred to use experienced subjects intensively trained to respond as fast as possible to simple stimuli in order to minimize the likelihood of any distortions (Danziger, 1980).^3^ Scientific self-observation (German: *Selbstbeobachtung*) would only be possible under such strict and simplified experimental conditions; otherwise there were only inner perception (*innere Wahrnehmung*) beyond the purview of science generally and psychological science in particular (Wundt, 1888).

The problem of introspection and the idea of the trained subject will also play a major role in the next section, but first the opportunity should be taken to contrast the positions summarized thus far with the behavioristic research program that would dominate psychology for decades after Wundt. In his seminal programmatic paper, John B. Watson was very skeptical of both investigating consciousness in general and the introspective method in particular (Watson, 1913). He saw the latter as “mental gymnastics” and found that it had “something esoteric.” Terms like “feeling” had no clear meaning–and therefore no place in science, just like consciousness at large. Psychology should, like other natural sciences, only deal with that which is objectively measurable. For Watson’s psychology this was only *behavior*. Although Burrhus F. Skinner (1904–1990), another famous behaviorist, expressed a less radical view about consciousness, he was also very skeptical of the place of “mental vocabulary” in science, particularly psychological science (Skinner, 1971), anticipating the philosophical position of *eliminative materialism* that emerged a little later (Churchland, 1981).

We have seen in this section that the idea of the “hard problem” of consciousness can be traced back until at least the 18th century. However, the view that consciousness is impossible or at least hard to explain mechanistically or reductively rather seems to be based on an appeal to intuition or imagination than on strict scientific reasoning; the arguments thus amount to a negative/skeptical stance and fall short of providing a strong positive reason for why the problem should be impossible to solve. Wundt described experimental conditions under which at least some perceptions could be observed scientifically, while Watson wanted to restrict science to the study of behavior. Both the problem of introspection and the critique that the meaning of certain vocabulary is unclear are still relevant today, even though consciousness has now become an accepted and even quite successful research domain. However, recent scientific reviews introduced above show that there’s still no agreement on either the *explanandum* or the *explanans* of consciousness research. We cannot ask Leibniz, Du Bois-Reymond, Wundt, Watson, or Skinner for their views on the present research; but we can discuss in the next section whether meditation research is a promising way to deal with the “hard problem.”

## Neurophenomenology and Meditation Research

Neuropsychological research on meditation has become so common that some actually already warn us to “mind the hype” (Komjathy, 2017; Van Dam et al., 2018) or its possible negative effects (Cebolla et al., 2017; Schlosser et al., 2019). However, for the purpose of this article we need not address whether meditation or its “mindfulness” component really has the health benefits so many now have come to believe. Our interest here is twofold: First, can experienced meditators produce conscious states with sufficient stability to solve the problem of introspection; and second, could subsequent research overcome the “hard problem” of consciousness?

Traditionally, an explanation of conscious experience was attempted by *phenomenological psychologists*. In particular Edmund Husserl (1859–1938) already distinguished first-person descriptions of lived experience from investigating consciousness within an empirical science (Husserl, 1911/1965).^4^ We addressed the problem of introspection in the previous section, particularly that consciousness is constantly changing. Discussing the neurophenomenological program originally developed by Varela from a present perspective, Berkovich-Ohana et al. (2020) recently acknowledged this very problem: “As lived experience is a constantly changing, multi-layered and highly complex flux, its exploration is challenging”. Similar to Wundt, Varela also emphasized the importance of developing certain skills for the experimental subject in consciousness research (Varela, 1996). As will be shown in the remainder of this section, meditation seems to be a promising technique to achieve precisely that.

While there are too many ways of practicing meditation to address here, recent and influential reviews generally distinguish three major kinds: attentional, constructive, and deconstructive (Dahl et al., 2015; Lutz et al., 2015). The first aims at sustaining attention on a certain object or process; the second at achieving a certain psychological state (e.g., compassion); and the third at getting rid of certain features of psychological processes that may be disturbing.^5^ The first and the third are most relevant for the purpose of this paper. There’s now a general consensus that sustained attention facilitates not only meditation, but also introspectively investigating consciousness (Dahl et al., 2015; Lutz et al., 2015; Berkovich-Ohana et al., 2020). For example, Lutz et al. (2015) described how practicing continuous attention on one’s psychological processes can lead to *dereification* (sometimes also called *cognitive defusion*), a state where one perceives emotions and thoughts without identifying with them as *my thoughts* or one realizes that there is anger without interpreting that *I am angry*. The latter also exemplify the deconstructive aspect of meditation.^6^ In that sense, (experienced) meditators would become more neutral observers of their own psychological processes. Sustained attention and a decrease of distractions (e.g., mind-wandering, rumination) would facilitate stable consciousness of a certain object or process–or perhaps even a state of *pure consciousness* without any identification or *consciousness of* something (Lutz et al., 2015; see also Metzinger, 2020). This skill of (very) experienced meditators seems, at least partially, to overcome the problem of introspection and meet Wundt’s (1888) requirements of scientific observation. Here we should distinguish two aspects of meditation which are independently, but complementarily related to introspection: The first is the attentional/mindfulness component that simply allows subjects to attend better to their psychological processes; the second are particular states of consciousness (arguably) only occurring in deep meditation, such as the example of pure consciousness addressed below. Regarding the former, this perspective aims at encouraging such approaches in phenomenological psychology; with respect to the latter, it serves as an example for how to solve the problem of introspection.

Many studies and reviews already described brain processes and areas related to meditation (e.g., Fox et al., 2016). With respect to the “hard problem,” however, it is more informative for the present purpose to look at one such particular study in more detail. Winter et al. (2020) used neuroimaging (EEG and fMRI) to investigate a (very) experienced meditator with more than 50,000 h of practice, sometimes up to 12 h of formal meditation per day. This subject was allegedly able to get into a state of pure consciousness–or content-free awareness–as described above. The meditator reported having achieved a state with “no awareness of any mental content or any sensory event, including the noise of the MRI scanner” and “no experience of self, time, or space of any kind whatsoever” under experimental conditions while being awake the whole time (Winter et al., 2020: 4).^7^ Summarized briefly, the authors report that this state was correlated with a sharp decrease in EEG alpha power and a decoupling between the dorsal attention network and the sensory cortex in the brain. This would be consistent with neural markers of sensory disconnection and a state of disconnected consciousness as reported in previous literature.

What does this mean with respect to the “hard problem”? We may recall that Leibniz invited us to imagine walking around in the meditator’s brain as if it were a mill and that du Bois-Reymond suggested that we may have all scientific knowledge of the meditator’s neural processes. Even though Winter et al. (2020) combined different neuroscientific methods to investigate a subject allegedly successful in producing a very stable conscious state, we still seem far away from the preconditions of Leibniz’s or du Bois-Reymond’s thought experiment. However, the present situation lets us draw at least some preliminary conclusion: First, investigating advanced meditators appears to be a promising experimental paradigm for empirical consciousness research going beyond what deemed Wundt possible (and even more so Watson or Skinner); second, even under such controlled conditions, first-person knowledge about the meditator’s phenomenology seems to be a prerequisite for interpreting the neuroscientific data; third, this interpretation requires background knowledge of how the brain works more generally; fourth, when these conditions are met, a plausibility check becomes possible to evaluate the first-person account in the light of third-person data–or the other way around. This could also mean that, in the long run, when meditation becomes understood better neuroscientifically, this knowledge might be used to guide meditators. Theoretically interesting cases would occur when, unlike in the study presently discussed, the first- and third-person accounts were *incongruent* (e.g., when a meditator’s alleged state of pure awareness looked neurally like a state of rumination, or *vice versa*; see also Schleim and Roiser, 2009; Schleim, 2018). But for the time being it seems justified to conclude that it is not possible, on the basis of what was discussed above, to assess how hard the “hard problem” really is. Instead, in line with the neurophenomenological research program (Varela, 1996; Berkovich-Ohana et al., 2020) the first- and third-person perspectives have the potential to inform each other. In particular, knowledge gained in such investigation could be used to design follow-up studies, which has been coined phenomenological “front-loading” before (Gallagher, 2003; Gallagher and Sørensen, 2006). Finally, assuming that meditators discuss previously unknown experiences with their teachers or with researchers employing specialized questionnaires (e.g., Wallace, 2007; Gamma and Metzinger, 2021), neurophenomenology could also include the second-person perspective.

## Summary and Outlook

Both consciousness and its “hard problem” have a long tradition in philosophy and science. The behaviorists’ views turned out to be too extreme and pessimistic, partially thanks to advances in psychology and neuroscience allowing researchers to look into the “black box” of the nervous system and the brain; but also Wundt’s views on what is experimentally possible seem too limited from a present perspective. His idea to use trained subjects undergoes a revival in the form of investigating long-term meditators presently.^8^ This kind of research also promises to combine first-, second-, and third-person approaches to study consciousness, without any of them making the others obsolete. Disagreement on the *explanandum* and *explanans* of consciousness research also illustrates, though, unresolved foundational issues. In line with Leibniz’s and du Bois-Reymond’s train of thought one can still question what the total knowledge of the mechanism underlying consciousness in general or meditative experiences in particular would be knowledge *of*. Therefore, finding minimally sufficient neural correlates of consciousness may primarily answer neuroscientific questions, without solving the “hard problem” as a whole. But is this actually important in general–or only interesting from a naturalistic point of view concerned with the completeness of natural science? New research on mechanisms also illustrates how scientific explanations can be *integrative* and combine different levels of description without automatically replacing or reducing them (Machamer et al., 2000; Craver, 2007). This perspective article suggested a few preliminary answers and tried to illustrate the diversity of available methods and paradigms to study consciousness as well as the continuation of phenomenological psychology in neurophenomenology. The present situation of consciousness research thus promises many more interesting findings, with research on long-term meditators (allegedly) producing stable states of consciousness being a particularly interesting path to follow.

## Data Availability Statement

The original contributions presented in the study are included in the article/supplementary material, further inquiries can be directed to the corresponding author.

## Author Contributions

SS conceived and wrote the whole manuscript, contributed to the article, and approved the submitted version.

## Conflict of Interest

The author declares that the research was conducted in the absence of any commercial or financial relationships that could be construed as a potential conflict of interest.

## Publisher’s Note

All claims expressed in this article are solely those of the authors and do not necessarily represent those of their affiliated organizations, or those of the publisher, the editors and the reviewers. Any product that may be evaluated in this article, or claim that may be made by its manufacturer, is not guaranteed or endorsed by the publisher.

## Footnotes

1 As is so often the case in philosophy, one could trace the origins of this argument even back to Greek antiquity: Plato described in *Phaido* how Socrates, shortly before executing his death sentence, discussed the materialistic philosophy of Anaxagoras. According to materialism, Socrates argued, a valid answer to the question why he is in prison would be that his bones and tendons moved such-and-such to transport his body there (*Phaido*, 98c–e; see also Polak, 1936). But this would miss the point of the question altogether (i.e., that he accepted the sentence of the Court of Athens instead of fleeing from the city). While this is obviously not exactly the same as the “hard problem” of consciousness, it already illustrates the competition between first- and third-person perspectives, intentional and naturalistic explanations, or the humanities and the natural sciences that will be central in the remainder of this article.

2 The article has 4,686 Google Scholar citations as of May 1, 2022.

3 In Danziger’s useful history of introspection in psychology, other approaches–like that of the Würzburg School–are discussed as well. Doing justice to these or contemporary approaches like micro-phenomenology (e.g., Petitmengin et al., 2019) goes beyond the scope of this perspective.

4 It is a matter of debate whether and to what extent Husserl’s method can be described as *introspection*. The answer is not central for my analysis. In line with Berkovich-Ohana et al.’ (2020) discussion which I rely on in what follows, Gutland discussed this in detail and answered the question affirmatively, applying Schwitzgebel’s criteria for introspection (Schwitzgebel, 2016; Gutland, 2018).

5 Constructive kinds of meditation not further addressed in this article are often exercised in the context of healthcare, wellness, or the ethics of particular spiritual traditions such as Buddhism (Wallace, 2007; Federman, 2021; Schmalzl et al., 2021).

6 While this perspective does not specifically address Husserl’s phenomenological approach, the striking similarity between attentional meditation (or mindfulness, to use the popular term) as described above and Husserl’s *epoché* should not be neglected; further similarities between deconstruction/dereification and Husserl’s noema or phenomenological reduction (Gutland, 2018) should be pursued in more detail elsewhere.

7 Some question the possibility of content-free awareness (consciousness without content) or pure consciousness. Here I would like to refer the interested reader to the discussion by Metzinger (2020) and Winter et al. (2020), with further references mentioned therein.

8 Not pursued here are transcultural perspectives related to meditation, such as the perception of classic Asian texts, the reinterpretation, or decontextualization of meditative practices by scholars in the West (Komjathy, 2017; Metzinger, 2020).
